# The new frontier of newborn screening: integrating genomics, metabolomics, and data science for inborn errors of metabolism

**DOI:** 10.1186/s13023-026-04513-0

**Published:** 2026-07-24

**Authors:** Muhammad Wasim, Yajun Wang, Guoda Ma

**Affiliations:** 1 Maternal and Children’s Health Research Institute, Shunde Women and Children’s Hospital, Guangdong Medical University, Foshan, 528300 China; 2 Department of Pediatrics, Shunde Women and Children Hospital, Guangdong Medical University, Foshan, 528300 China

**Keywords:** Newborn screening, Inborn errors of metabolism, Metabolomics, Precision medicine, Tandem mass spectrometry

## Abstract

Newborn screening (NBS) for inborn errors of metabolism (IEMs) represents one of the most successful public health initiatives of the past century. Originating with the simple bacterial inhibition assay for phenylketonuria, the field was revolutionized by tandem mass spectrometry (MS/MS). Today, we stand at the precipice of a second, more profound revolution driven by the convergence of high-throughput genomics, next-generation metabolomics, and advanced data science. This review articulates this paradigm shift, detailing how MS/MS is being augmented by targeted DNA analysis and how whole genome sequencing and untargeted metabolomics, integrated with artificial intelligence, are expanding the detectable universe of disorders and enabling precision interventions. However, this powerful multi-omic approach raises critical challenges: incomplete penetrance, overdiagnosis, economic burden, psychosocial impacts, algorithm biases, and the genomic data gap that exacerbates health disparities. We critically examine the practical limitations of AI, the lack of evidence for cost-effectiveness, the slow pace of database updates, and the need for short-term reanalysis systems to “rescue” cases left undiagnosed. Global policy variations and data-sharing governance frameworks are also addressed. We conclude that the future of NBS lies not in sequential technology replacement, but in the responsible creation of a sophisticated, multi-omic biomedical information system to transform NBS into a foundational pillar of precision pediatric medicine.

**Graphical Abstract:**

**Figure:**
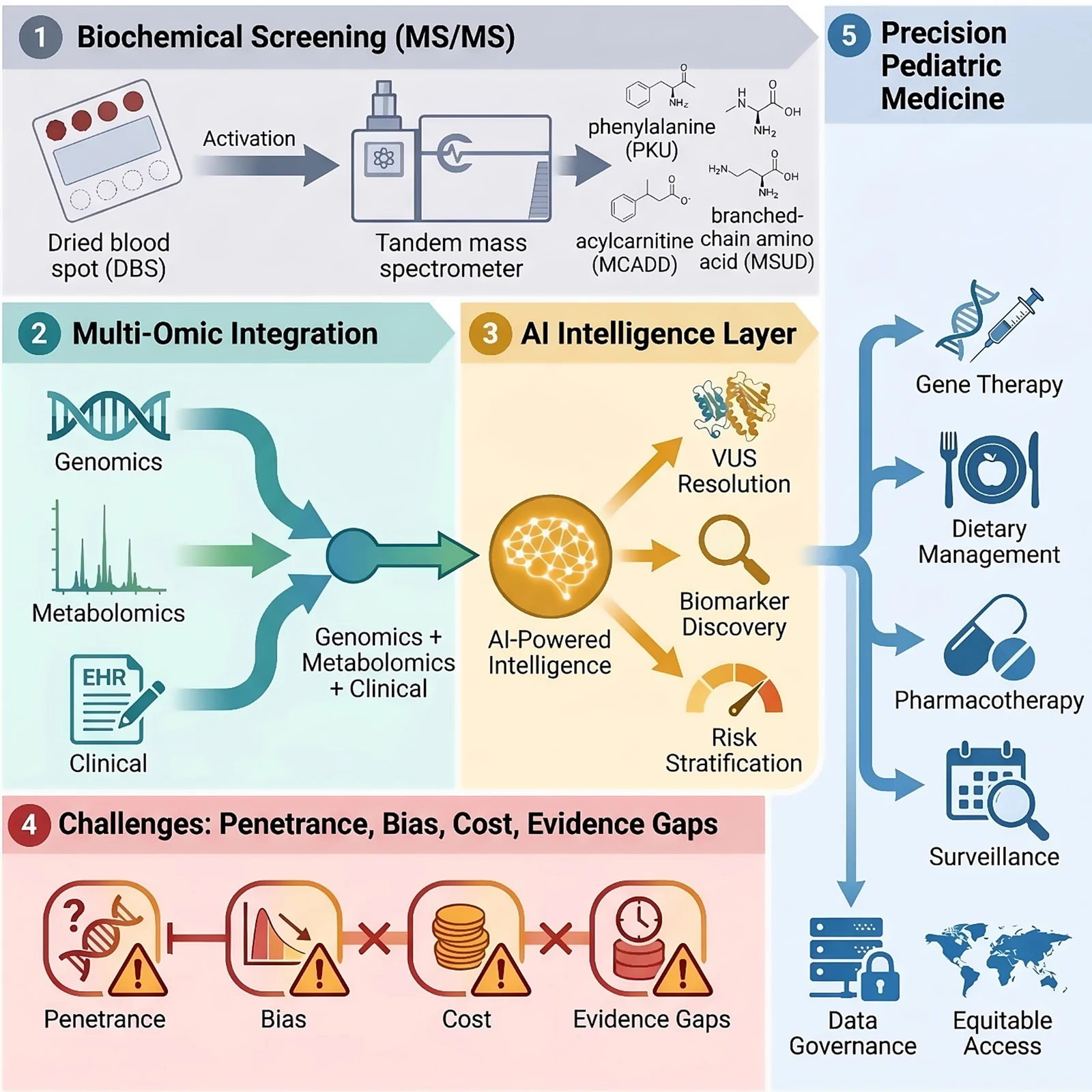

## Clinical Trial Number

Not applicable.

## Introduction: from a single test to a complex information ecosystem

Inborn errors of metabolism (IEMs) are a vast, genetically heterogeneous group of disorders caused by defects in enzymatic pathways responsible for the synthesis, breakdown, or transport of biochemical substrates [1, 2]. Individually rare, they collectively represent a significant cause of pediatric morbidity and mortality, often presenting with catastrophic, irreversible neurological or metabolic deterioration in the first days or weeks of life [3]. The central premise of newborn screening (NBS) is that pre-symptomatic detection of these disorders enables rapid intervention, dietary modification, cofactor supplementation, or specific pharmacotherapy, to prevent disability and death [4–6].

The history of NBS for IEMs is a history of technological advancement driving public health impact. The 1963 Guthrie test for PKU inaugurated the era [7], followed by incremental additions using bacterial, fluorescent, or immunoassay techniques [8]. The pivotal breakthrough came in the 1990s with the application of tandem mass spectrometry (MS/MS) to dried blood spots (DBS), allowing the simultaneous quantification of dozens of amino acids and acylcarnitines [9–11]. This “one test, many diseases” model led to a rapid global expansion of NBS panels, making disorders like medium-chain acyl-CoA dehydrogenase deficiency (MCADD) and maple syrup urine disease (MSUD) detectable and manageable [12, 13].

In the NBS literature, tandem mass spectrometry (MS/MS) is commonly employed in a targeted metabolomics capacity, the quantitative measurement of a predefined panel of amino acids and acylcarnitines with well-established clinical associations and reference ranges [14–17]. This approach is hypothesis-driven, requiring prior knowledge of diagnostically informative metabolites. In contrast, next-generation or untargeted metabolomics utilizes high-resolution mass spectrometry (HR-MS) to comprehensively profile thousands of small molecules, including lipids, nucleotides, and bile acids, without pre-selection, enabling biomarker discovery and pattern recognition for complex or ultra-rare disorders [18, 19]. While both modalities fall under the broader metabolomics umbrella, their analytical objectives, technical requirements, and roles within the NBS ecosystem are fundamentally distinct [20, 21].

Today, the field is undergoing a second, more complex transformation. The classic Wilson & Jungner criteria for screening focused on a well-understood disorder with an acceptable treatment and a cost-effective test are being stretched by new realities [22]. The development of life-saving but extraordinarily expensive therapies (e.g., gene therapy for spinal muscular atrophy and metachromatic leukodystrophy) creates a powerful impetus for early detection [23]. While our group has previously published on the diagnosis of treatable IEMs in settings lacking NBS program [24–39], the landscape is now being transformed by the “omics” revolution. The “omics” revolution offers tools of unprecedented power and complexity. The core thesis of this review is that the integration of genomics, next-generation metabolomics, and data science is creating a new frontier for NBS [40, 41]. This integration is not merely additive; it is synergistic, transforming NBS from a biochemical testing program into a dynamic, learning biomedical information system.

Hence, this narrative review synthesizes the current landscape of NBS for IEMs, a comprehensive literature search was conducted using PubMed, Scopus, and Web of Science databases for articles published between January 2000 and June 2026. The search strategy combined keywords including “newborn screening,” “inborn errors of metabolism,” “tandem mass spectrometry,” “whole genome sequencing,” “metabolomics,” “artificial intelligence,” “machine learning,” and “ethical challenges.” Additional references were identified through manual screening of bibliographies from key review articles and guideline papers. Inclusion criteria encompassed peer-reviewed original research, systematic reviews, meta-analyses, clinical practice guidelines, and position statements from major professional organizations. Priority was given to landmark studies that have shaped the field, and recent technological advances. Evidence synthesis focused on identifying thematic convergences across technological, clinical, and ethical dimensions, employing a conceptual framework approach to organize findings into integrated technological pillars, therapeutic categories, and implementation challenges.

Moreover, Table 1 explains the evolution of NBS technology, highlighting the paradigm shift from single-analyte biochemical assays to today’s multi-omic integration. This trajectory underscores the increasing diagnostic power, while revealing inherent limitations such as false positives or variant interpretation challenges that each new era must address.

**Table 1:** Evolution of NBS technologies for IEMs: capabilities and limitations

Technology Era	Key Methodology	Example Disorders Detected	Key Advantages	Primary Limitations	References
Classic (1960s-1990s)	Bacterial inhibition assay, fluorimetry, immunoassays.	Phenylketonuria (PKU), Congenital hypothyroidism (CH), Galactosemia.	Simple, low-cost, proven for high-prevalence disorders.	One test per disorder, low throughput, limited menu.	[7, 42]
Tandem Mass Spectrometry (MS/MS) Era (1990s-Present)	Multiplexed analysis of amino acids & acylcarnitines from DBS.	MCADD, MSUD, Propionic Acidemia, GA-I, PKU, Tyrosinemias.	High-throughput, “one test for many diseases,” quantitative, excellent sensitivity/specificity.	Predefined analyte panel, false positives, ambiguous profiles, cannot detect all IEM types.	[10, 11, 43]
Integrated Genomics Era (2010s-Present)	2nd-tier targeted DNA panels, NGS.	Confirmation & subtyping of MS/MS findings (e.g., *SLC22A5* for CUD), SMA (via *SMN1* deletion).	Reduces false positives, provides definitive diagnosis, and enables carrier detection.	Adds cost/complexity, limited to known genes, requires expert interpretation.	[8, 44–46]
Multi-Omic & AI Frontier (Emerging)	Untargeted HR-MS metabolomics, WGS/WES, AI/ML integration.	Discovery of novel IEMs, ultra-rare disorders, complex phenotypes, polygenic risk.	Holistic profiling, biomarker discovery, powerful data integration, predictive analytics.	Immense data volume, high cost, major VUS/interpretation challenges, ethical/ELSI hurdles.	[47–49]

AI, Artificial Intelligence; CH, Congenital Hypothyroidism; CUD, Carnitine Uptake Defect; DBS, Dried Blood Spot; ELSI, Ethical, Legal, and Social Implications; GA-I, Glutaric Aciduria Type I; HR-MS, High-Resolution Mass Spectrometry; IEMs, Inborn Errors of Metabolism; MCADD, Medium-Chain Acyl-CoA Dehydrogenase Deficiency; ML, Machine Learning; MS/MS, Tandem Mass Spectrometry; MSUD, Maple Syrup Urine Disease; NBS, Newborn Screening; NGS, Next-Generation Sequencing; PKU, Phenylketonuria; SMA, Spinal Muscular Atrophy; VUS, Variant of Uncertain Significance; WES, Whole Exome Sequencing; WGS, Whole Genome Sequencing

## The foundational pillar and its evolution: tandem mass spectrometry and beyond

### The workhorse: tandem mass spectrometry (MS/MS)

MS/MS remains the unchallenged technological backbone of IEM screening. Its principle allows for highly specific and sensitive multiplexed analysis from a single 3.2 mm DBS punch [50–52]. Standard panels quantify key metabolites like amino acids: elevated phenylalanine (PKU), leucine/isoleucine (MSUD), tyrosine (Tyrosinemia type I), and acylcarnitines: Specific profiles indicate fatty acid oxidation disorders (e.g., elevated C8, C10, C10:1 for MCADD) and organic acidemias (e.g., elevated C3 for propionic acidemia, C5DC for glutaric aciduria type I) [53–55]. The success of MS/MS is undeniable, but it has inherent limitations: it measures a predefined set of analytes, can yield false positives due to non-pathological factors (prematurity, parental nutrition), and often provides ambiguous patterns requiring follow-up testing [56–58].

### Second-tier molecular testing: refining specificity

To address the false-positive challenge, second-tier DNA testing has become a standard adjunct. When MS/MS reveals an abnormal pattern suggestive of a specific disorder, DNA can be extracted from the residual DBS and analyzed via targeted next-generation sequencing (NGS) panels for genes associated with that metabolic pathway [59, 60]. For example, an elevated C0 (free carnitine) and specific acylcarnitine profile suggestive of primary carnitine uptake defect (due to SLC22A5 mutations) can be rapidly confirmed genetically [61, 62]. This approach drastically reduces the number of infants requiring stressful clinical recall, accelerates definitive diagnosis, and alleviates parental anxiety. It represents the first, now-routine, integration of genomics into the NBS workflow [63–65].

### Next-generation metabolomics: expanding the detectable universe

While MS/MS is a form of metabolomics, “next-generation” or “untargeted” metabolomics represents a qualitative leap. Using high-resolution mass spectrometry (HR-MS) or nuclear magnetic resonance (NMR) spectroscopy, these platforms can detect thousands of small molecules in a DBS without a pre-defined target list [20, 66]. This capability is transformative for two reasons: 1) Biomarker Discovery; For ultra-rare or novel IEMs lacking known biomarkers, untargeted metabolomics can identify unique metabolic signatures by comparing profiles from affected and unaffected infants, and 2) Pattern Recognition for Complex Diagnoses; Some IEMs present with subtle or overlapping MS/MS patterns [67, 68]. Machine learning (ML) algorithms applied to broad metabolomic profiles can improve discrimination. For instance, differentiating between subtypes of urea cycle disorders or mitochondrial diseases may be enhanced by analyzing a wider array of metabolites, including lipids, nucleotides, and bile acids. The integration of broad-spectrum metabolomic data moves NBS from a hypothesis-driven towards a discovery-oriented system [69–71].

**Fig. 1:**
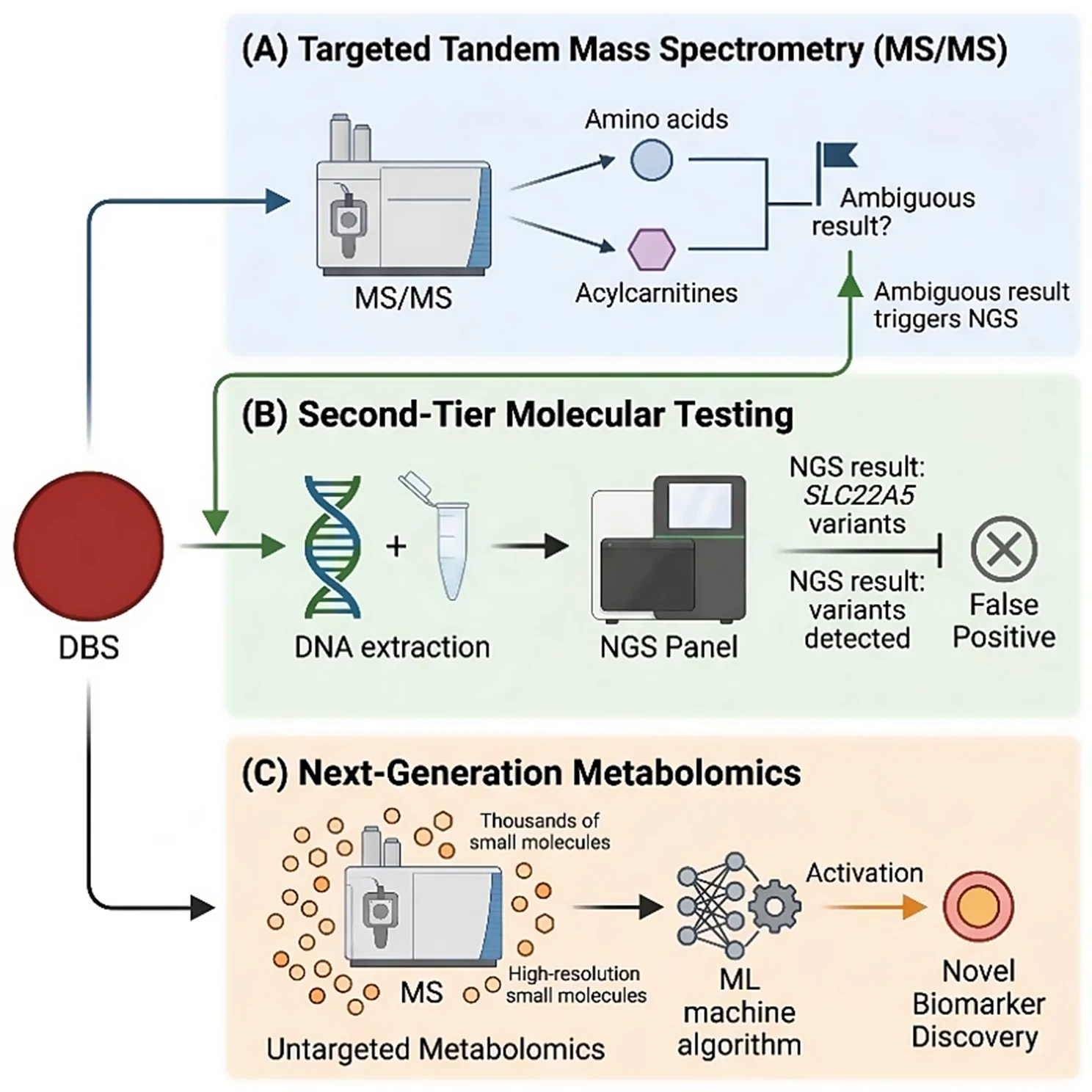
From targeted quantification to discovery-oriented profiling: the integrated technological workflow of modern newborn screening. The schematic illustrates the three complementary pillars of contemporary NBS. (**A**) Targeted Tandem Mass Spectrometry (MS/MS): The foundational workhorse that quantifies a predefined panel of amino acids and acylcarnitines from a dried blood spot (DBS) to flag disorders like PKU and MCADD, though it can yield ambiguous results. (**B**) Second-Tier Molecular Testing: A reflexive genomic step triggered by an ambiguous MS/MS result. DNA extracted from the same DBS is analyzed via targeted next-generation sequencing (NGS) gene panels to provide a definitive molecular diagnosis (e.g., confirming SLC22A5 variants in primary carnitine uptake defect), thereby reducing false positives. (**C**) Next-Generation Metabolomics: An untargeted, discovery-oriented platform using high-resolution mass spectrometry to profile thousands of small molecules. This approach, often aided by ML, enables the discovery of novel biomarkers for ultra-rare IEMs and improves pattern recognition for disorders with complex, overlapping biochemical phenotypes. Abbreviations: DBS, Dried Blood Spot; NGS, Next-Generation Sequencing; ML, Machine Learning; IEM, Inborn Error of Metabolism. Image was created using BioRender, in conjunction with Microsoft PowerPoint for final assembly and labeling

Moreover, Figure 1 shows the foundational pillar of IEM screening is MS/MS, which provides targeted, high-throughput metabolite analysis. This core method is now augmented by second-tier molecular testing for definitive genetic confirmation and next-generation, untargeted metabolomics for biomarker discovery. This integrated, multi-layered approach refines specificity, reduces false positives, and expands the detectable universe of metabolic disorders.

## The disruptive integrator: genomic sequencing in the NBS context

The prospect of applying whole exome sequencing (WES) or whole genome sequencing (WGS) as a primary or universal screening tool is the most debated topic in modern NBS.

### Potential and promise

WGS offers a paradigm shift in potential for NBS. Its comprehensiveness could detect a vast array of genetic disorders at birth, from neurodevelopmental to cardiometabolic conditions, providing definitive molecular diagnoses that bypass lengthy diagnostic odysseys [72, 73]. Crucially, WGS acts as an enabler for “therapies on demand,” making early identification imperative as gene-targeted treatments like those for spinal muscular atrophy proliferate [74–76]. Furthermore, it can yield valuable incidental information, such as parental carrier status or pharmacogenomic data to guide future drug safety [77].

### Pilot programs and early data

Projects like the BabySeq Project (USA) and the Genomics England Newborn Genomes Programme (NewbornGenomes Programme | Genomics England) are generating crucial data [78, 79]. Early results confirm WGS can identify risk for childhood-onset disorders beyond standard NBS, but they also quantify the challenges: a significant proportion of results are variants of uncertain significance (VUS), and actionable findings for disorders with no clear neonatal intervention pose ethical dilemmas [80–82].

### The central challenge: the VUS tsunami and actionability

The fundamental limitation is not technical, but interpretative. For every clear-cut pathogenic variant in a gene like *PAH* (PKU), WGS will reveal several VUSs in other metabolic genes [83, 84]. Determining which represent true disease risk versus benign population variation is immensely difficult in an asymptomatic newborn [85, 86]. The ACMG Secondary findings list offers guidance for reporting incidental adult-onset conditions, but a consistent framework for actionable pediatric-only findings from population screening is lacking. This creates a risk of iatrogenic harm through over-medicalization of healthy infants [87, 88].

### Critical limitations of WGS in NBS

Beyond variant interpretation challenges, WGS-based screening faces additional fundamental limitations. Many metabolic disorders exhibit incomplete penetrance and variable expressivity [89], meaning that a pathogenic genotype does not reliably predict disease onset, severity, or even clinical manifestation [90]. The detection of pathogenic variants for untreatable or poorly treatable conditions raises ethical concerns about screening without therapeutic benefit [91]. The economic burden of universal WGS, including downstream diagnostic cascades and lifelong surveillance [92], may divert resources from proven interventions, yet its cost-effectiveness in a NBS context remains unknown. Furthermore, the long-term psychosocial consequences of identifying infants as genetically “at-risk” can manifest as chronic parental anxiety and the “patient-in-waiting” phenomenon [91]. Finally, the risk of overdiagnosis - identifying variants that would never cause clinical disease - can lead to unnecessary interventions and iatrogenic harm [90]. These limitations demand that the implementation of genomic screening proceeds with caution, robust counseling infrastructure, and continuous evaluation of outcomes to ensure that benefits outweigh risks for all newborns [93].

### Moving beyond monogenicity: the role of genetic background and polygenic risk scores

Although most disorders currently targeted by NBS are considered monogenic, emerging evidence demonstrates that the clinical manifestation of a pathogenic variant is often significantly modulated by the individual’s broader genetic background. Incomplete penetrance and variable expressivity are increasingly recognized as the rule rather than the exception in Mendelian disease [94]. Mounting evidence suggests that common polygenic variation can independently influence the phenotype among carriers of rare monogenic variants. In 80,928 individuals, polygenic background altered disease risk in monogenic variant carriers, with probabilities by age 75 ranging 17–78% (coronary artery disease) and 13–76% (breast cancer) [95]. These findings have profound implications for genomic NBS. Binary variant classification is insufficient for genomic NBS. Polygenic risk scores (PRS) could provide more accurate risk estimates, distinguishing severe from asymptomatic cases, refining counseling and surveillance [96, 97]. However, PRS implementation requires ancestry-diverse reference datasets and validated frameworks [98, 99].

### Clinical classification of screenable disorders and the role of genetic and environmental modifiers

A critical step in the responsible implementation of genomic NBS is the systematic classification of all potentially screenable disorders according to key clinical parameters [100]. This classification enables prioritization of conditions with impactful early intervention while identifying areas lacking effective therapies for R&D focus [101]. However, the treatable-untreatable distinction is increasingly blurred, as many “untreatable” disorders may still benefit from supportive care or emerging therapies [102]. For instance, a DNA-first systematic evaluation identified 95 inherited metabolic disorders (IMDs) as eligible for NBS based on treatability criteria, including 58 conditions beyond those currently detected by biomarker-based screening [101]. Conversely, disorders of profound severity with curative treatments available represent the highest priority for inclusion, whereas conditions with milder phenotypes, lower penetrance, or no current treatment require more nuanced consideration [100].

Beyond treatability, the phenotypic variability in IEMs (asymptomatic to severe) necessitates understanding disease expression modulators [103]. The “one gene-one disease” paradigm has given way to recognition that environmental, epigenetic, microbiome, and genetic factors shape clinical phenotypes [103]. Severity and onset are modulated by residual protein activity influenced by genetic and environmental factors [104]. For example, a large-scale population study of >900,000 adults found that only 33% of individuals carrying (likely) pathogenic TSC1/TSC2 variants had a recorded diagnosis of tuberous sclerosis complex, challenging the assumption of near-complete penetrance and highlighting the contribution of genetic background and environmental modifiers [105]. Determining whether variability in severity or onset arises from the variant, genetic background (modifier genes, polygenic risk), or environmental factors (diet, infections, medications) is essential for NBS counseling, surveillance, and clinical decisions. This knowledge distinguishes cases needing early intervention from those appropriate for watchful waiting, preventing unnecessary medicalization.

## The essential interpreter: data science, AI, and ML

The integration of multi-omic data generates a complexity that surpasses human analytical capacity. This is where advanced data science becomes indispensable.

### Artificial intelligence (AI) for pattern recognition and risk stratification

Artificial intelligence (AI), especially deep learning, is becoming essential for interpreting the massive, integrated datasets of modern NBS [106, 107]. These models are trained on multi-omic and clinical data, these models triage results for urgent cases, discover novel biomarker patterns, and integrate electronic health records to refine individualized risk predictions and diagnoses [69, 108].

### AI for genomic interpretation

Advanced AI tools are crucial for resolving the central challenge of variant interpretation. Systems like AlphaMissense predict the pathogenicity of missense mutations by analyzing protein structure and evolutionary data, potentially reclassifying thousands of VUS in IEM genes [109–111]. Furthermore, natural language processing algorithms can mine electronic health records to extract subtle phenotypic clues, enabling more accurate genotype-phenotype linking for precise diagnosis [112–115].

### The need for infrastructure and biobanks

The power of AI is contingent on large, high-quality, diverse training datasets. This underscores the critical need for national and international NBS data repositories and biobanks that link de-identified multi-omic data with long-term clinical follow-up. Initiatives like the INFORM-SDD registry for SMA are disease-specific models that need scaling to the entire NBS spectrum [116–118].

**Fig. 2:**
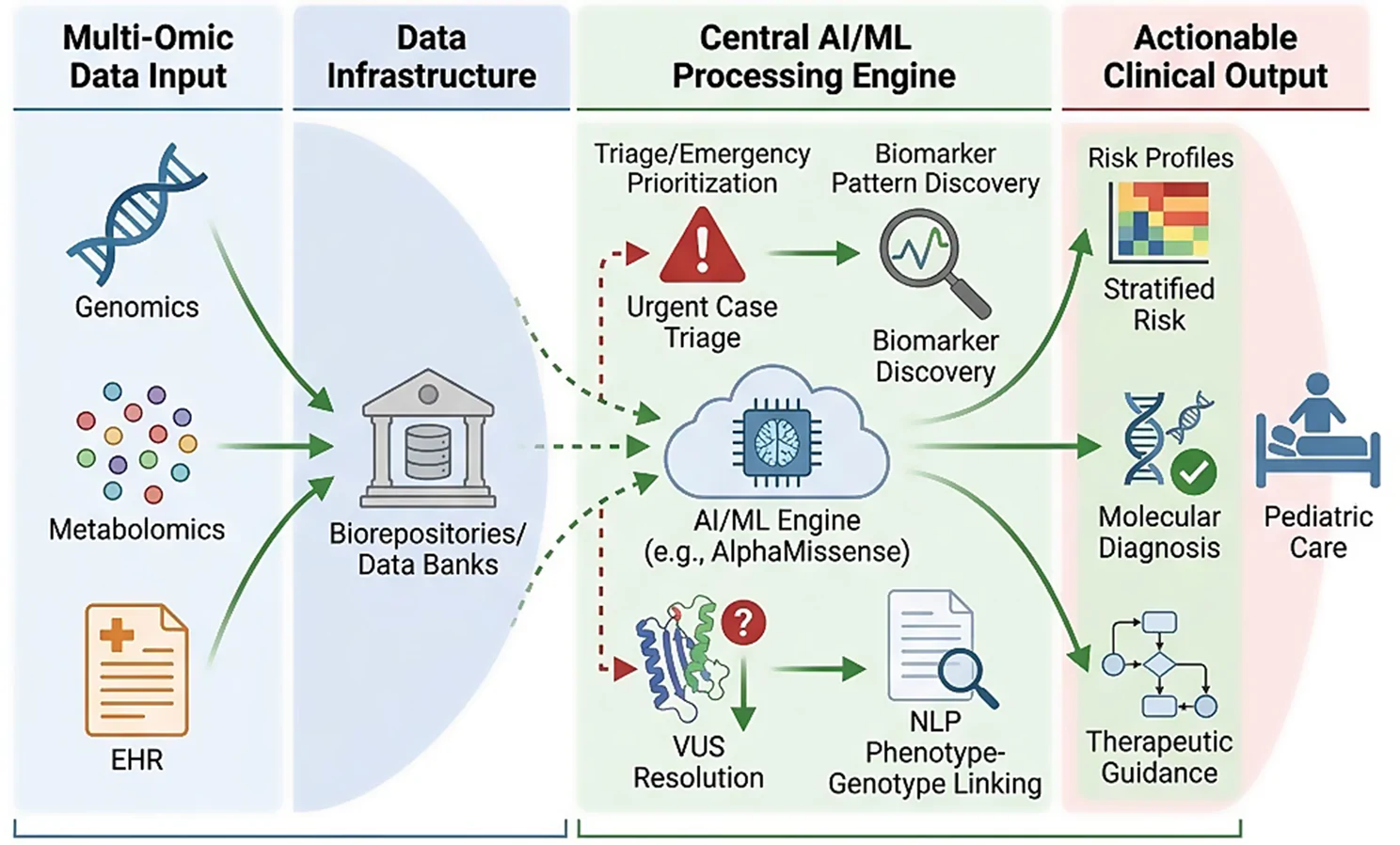
The AI-powered workflow: transforming multi-omic data into precision pediatric action. The schematic depicts the central role of artificial intelligence (AI) in advanced NBS. (1) Multi-Omic Data Input: Diverse data streams—genomic variants, complex metabolomic profiles, and clinical electronic health records—converge, creating an analytical challenge. (2) The AI Processing Engine: A ML system, exemplified by tools like AlphaMissense for variant interpretation, performs core functions: triaging results to prioritize urgent cases, discovering novel biomarker patterns, resolving variants of uncertain significance (VUS) using protein structural data, and mining clinical notes for phenotype-genotype linking via natural language processing (NLP). (3) Foundational Data Infrastructure: The power of AI is contingent on large-scale, linked, and diverse biorepositories and data banks that provide de-identified, longitudinal training data. (4) Actionable Clinical Output: The processed intelligence enables precise, individualized outputs, including stratified risk profiles, definitive molecular diagnoses, and guided therapeutic pathways for early intervention. The system underscores a transition from data complexity to preventative, precision medicine. Abbreviations: AI, Artificial Intelligence; ML, Machine Learning; VUS, Variant of Uncertain Significance; EHR, Electronic Health Record; NLP, Natural Language Processing. Image was created using BioRender, in conjunction with Microsoft PowerPoint for final assembly and labeling

The integration of genomics, metabolomics, and clinical data creates an interpretative challenge that surpasses human scale. AI and ML have thus become indispensable for transforming this data deluge into actionable clinical insights, from triaging results and resolving genetic variants to enabling precise, individualized risk prediction. Hence, Figure 2 illustrates AI as the central interpreter in the multi-omic NBS Ecosystem.

## Practical limitations and unresolved challenges of AI in NBS

Despite the considerable promise of AI and ML in NBS, the practical translation of these technologies into clinical and public health practice faces substantial and often underappreciated limitations that warrant careful consideration.

### Algorithmic bias and dataset imbalance

A fundamental concern is algorithmic bias, where AI models trained on non-representative datasets (overwhelmingly skewed toward majority groups by race, ethnicity, gender, and socioeconomic status) systematically underperform for underrepresented populations. For example, the FairSeg dataset comprises 76% white patients with only 24% Black and Asian patients, reducing model effectiveness for minorities and potentially exacerbating healthcare disparities [119–121]. In NBS, where early accurate detection is critical, biased AI models trained predominantly on European-ancestry data may miss or misclassify metabolic variants in non-European populations. The National Academies of Sciences, Engineering, and Medicine has called for research to ensure AI capabilities are applied to NBS “in a manner that maximizes benefits while minimizing risks” [122–125].

### Lack of transparency and the “Black Box” problem

Many advanced ML models, particularly deep learning architectures, operate as “black boxes” whose internal decision-making processes are opaque and difficult to interpret. This lack of transparency poses a significant challenge in medical applications, where clinicians and patients must trust and act upon algorithmic recommendations [126]. In NBS, where a false positive can cause significant parental anxiety and a false negative can result in catastrophic, irreversible disability, the inability to understand why an AI model made a particular prediction is a critical barrier to clinical adoption. While explainable AI (XAI) techniques offer a promising path forward, they must be coupled with domain expertise, rigorous validation, and transparent governance structures. However, a systematic review of the literature found that the application of XAI for ML methods in NBS has not been proposed in any identified study to date, representing a significant gap in the field [121, 125, 127–129].

### Regulatory oversight and clinical validation

The integration of AI into NBS raises complex regulatory questions. In the United States, the Food and Drug Administration (FDA) regulates AI/ML algorithms as medical devices when intended for diagnosis or treatment, employing a “total product lifecycle” approach covering data quality, algorithm transparency, and change management. However, the regulatory framework for AI in NBS remains nascent and ill-defined. Unlike traditional biochemical assays, AI models are subject to “model drift” (performance degradation over time due to changing demographics, laboratory practices, or disease prevalence) necessitating continuous validation, yet clear re-validation guidelines are lacking. As one analysis noted, “the illusion of safety” can arise when AI healthcare products are approved without adequate post-market surveillance [125, 130, 131].

### Limited generalizability across populations and settings

AI models trained on data from one population or healthcare setting frequently fail to generalize to others. This limitation is particularly acute in genomic medicine, where gold-standard genomic datasets severely under-represent non-European populations, leading to inequities and a limited understanding of human disease. As one review noted, addressing ancestry imbalance in genomic data “will require years of dedicated large-scale sequencing efforts”. Even with diverse training data, models may fail on novel or ultra-rare variants due to a lack of similar examples in the training data. In NBS, where many IEMs are individually rare, the challenge of generalizability is magnified. A model that performs excellently in one region or population may produce unreliable results when deployed elsewhere, necessitating external validation in diverse clinical environments [125, 130–134].

### Reproducibility concerns

The reproducibility crisis in biomedical AI represents a fundamental challenge to the clinical translation of these technologies. A 2021 analysis of 511 ML papers in health found low levels of dataset and code sharing, complicating replication efforts. Even when code is made publicly available, running it may not yield the same results due to the non-deterministic nature of learning models, variations in software systems, differences in hardware settings, data preprocessing variability, and the use of different optimizers [135]. As one critical review noted, “the rapidly growing complexity of these models compromises their interpretability, which currently impedes their successful and widespread clinical adoption”. For NBS programs that must make high-stakes decisions about infant health, the inability to independently verify and reproduce AI model predictions is a profound obstacle to implementation [125, 130, 131, 136, 137].

### Specific limitations of AlphaMissense and similar tools

Even state-of-the-art AI tools for variant interpretation have notable limitations. AlphaMissense, while achieving over 90% sensitivity and specificity in benchmarks, has been shown to have precision of only 32.9% and recall of 57.6% for expert-curated pathogenic variants in a cohort of 45 rare diseases [138]. As a large deep learning model, it lacks interpretability, does not assess the functional impact of variants, and provides pathogenicity scores that are not disease-specific [139]. Furthermore, AlphaMissense struggles to evaluate pathogenicity in intrinsically disordered regions (IDRs), resulting in unreliable gene-level essentiality scores for genes containing IDRs [138]. These limitations underscore that AI tools are adjuncts to, not replacements for, expert clinical interpretation, and highlight “ongoing challenges in clinical genetics” that demand “continued refinement of computational methods in variant pathogenicity prediction” [138].

## The challenge of evidence generation and knowledge translation in rare diseases

A critical challenge in genomic NBS is the profound disconnect between rapid data generation and slower evidence synthesis and database updating. This is particularly acute in rare diseases, where case scarcity, heterogeneity, and limited generalizability create a “knowledge chasm” that threatens screening validity and safety [140].

### The evidence generation gap

The systematic evaluation of NBS using WGS presents considerable challenges for policy advisors, not least given the logistics of synthesizing evidence across hundreds of individually rare conditions [140]. A landmark series of systematic reviews conducted for the UK National Institute for Health and Care Research (NIHR) evaluated the evidence base for WGS-based NBS across 200 conditions and found that current evidence synthesis methods are “neither feasible nor fruitful” for policy making [93]. A team of five reviewers would take over 20 years to conduct similar systematic reviews for 200 conditions. Critically, no studies were identified that addressed four of six core research questions including penetrance, accuracy, and harms of screening, for the five conditions selected for in-depth review. The reviewers concluded that “the evidence is not available to synthesize” and that implementation without substantial further research would “cause harm and uncertainty” [93, 140]. Compounding this is the generalizability gap. Evidence from symptomatic clinically detected cases is often not generalizable to screening populations, where disease spectra differ and less severe phenotypes predominate [140]. Thus, penetrance and treatment estimates from symptomatic cohorts may be inaccurate for asymptomatic newborns. As Mütze et al. noted, even established screening programs lack robust long-term evidence, with limitations including “incompletely understood natural history” and “unreliable early prediction of disease severity” [141].

### The database update problem

The challenge of evidence translation is exacerbated by slow updates in curated variant databases like ClinVar and HGMD. A comprehensive analysis revealed that ClinVar variants classified as Pathogenic/Likely Pathogenic are reclassified sixfold more often than HGMD variants, and 11-fold more often when considering reclassification towards reduced pathogenicity, reflecting differences in curation philosophies and update frequencies [142]. The practical implications are profound: ClinGen-VCEPs target re-evaluation of all Likely Pathogenic/VUS variants every two years, though some experts advocate shorter intervals given rapid molecular genetics advances [143]. However, reclassification intervals range from 8 days to 1.5 years, with half occurring ≥ 7 months after initial testing, during which clinical decisions may be based on outdated classifications with potentially serious consequences [140].

### Consequences for NBS

The implications for NBS are particularly concerning. Overloading the variant interpretation pipeline with VUS variants increases turnaround time and causes parental anxiety [93]. Providers are often surprised by VUS-to-pathogenic reclassifications when unaware of the variants. A study of VUS reclassification in neonatal genetic diseases found that reclassification clarified molecular diagnoses in 19 cases, increasing WES diagnostic rate from 30.2% to 33.0%, a modest but clinically meaningful improvement that would have been missed without proactive reanalysis [93]. This challenge is compounded by clinically identified variants often being novel to public databases: one study found 76 of 179 variants (42.5%) were novel to ClinVar, confirming that clinically identified variants represent a significant, under-utilized resource. This “siloed nature of clinical variants” limits translational research for rare disorders, particularly for rapid identification of research-eligible individuals [93].

### Toward a solution

Addressing these challenges requires a multi-pronged approach. First, there is an urgent need for mandatory long-term follow-up of NBS cohorts to generate the empirical evidence on penetrance, expressivity, and treatment outcomes that is currently lacking [141]. Second, interoperable patient registries are multi-purpose tools that could help close current knowledge gaps and inform further optimization of NBS strategy [141]. Third, proactive variant reclassification policies are essential to ensure that patients benefit from evolving knowledge. Finally, data sharing and communication must be strengthened, including sharing in-house databases among laboratories and uploading variants to public databases [93, 143].

## The expanding clinical landscape: from dietary management to gene therapy

The integration of new technologies is synergistic with the development of novel therapeutics, reshaping which disorders are considered “screenable” [144, 145].

- Category 1: Disorders Amenable to Dietary Therapy (The Classics): PKU, MSUD. NBS remains vital, with research on improved formulas and adjunct therapies (e.g., sapropterin, pegvaliase) [146, 147].
- Category 2: Disorders with Cofactor Therapy: Biotinidase deficiency, Pyridoxine-dependent epilepsy. NBS ensures immediate, life-saving treatment [28, 148].
- Category 3: Disorders with Enzyme Replacement Therapy (ERT): Pompe disease, MPS I. NBS is non-negotiable here. Outcomes are profoundly better when ERT is initiated pre-symptomatically, before irreversible cardiac or muscular damage occurs [149-151].
- Category 4: Disorders with Emerging Gene-Based Therapies (The New Vanguard): This category is the primary driver of NBS expansion [152, 153].

- Spinal Muscular Atrophy (SMA): DNA-based screening is now standard in many countries due to the efficacy of gene therapy [154–156].
- Metachromatic Leukodystrophy (MLD): Gene therapy is approved in the EU, making early detection via NBS (likely combining enzyme assay and arylsulfatase A (ARSA) gene sequencing) imperative [157–161].
- Adrenoleukodystrophy (ALD): Hematopoietic stem cell transplant is effective only if performed early. NBS (via C26:0 lysophosphatidylcholine assay) is now on the US Recommended Uniform Screening Panel (RUSP) [162, 163].

- Category 5: The Challenge of “Actionability”: Some severe IEMs (e.g., neonatal-onset urea cycle disorders, some mitochondrial disorders) still lack definitive therapies. Should we screen? The ethical calculus is shifting; identifying infants allows for prompt palliative care, genetic counseling, and enrollment in clinical trials, while also driving therapeutic research [5, 6, 44, 164–166]. Hence, Table 2 classifies screenable IEMs by pathophysiology and treatment, linking NBS rationale to therapeutic advancements, particularly gene-targeted therapies requiring pre-symptomatic detection

**Table 2:** Spectrum of screenable IEMs categorized by pathophysiology and treatment modality

Category & Rationale for NBS	Pathophysiological Mechanism	Representative Disorders	Primary Treatment Modality	Critical Role of Pre-Symptomatic Detection	Key References
**Dietary/Substrate Reduction** Prevents toxic accumulation.	Deficiency in amino acid or carbohydrate catabolism.	PKU, MSUD, Galactosemia.	Lifelong specialized diet (low Phe, low branched-chain AAs, lactose-free).	Prevents irreversible intellectual disability, metabolic crisis, and liver failure.	[167, 168]
**Cofactor/Vitamin Responsive** Enzyme function partially restored.	Defective apoenzyme with residual activity enhanced by high-dose cofactor.	Biotinidase Deficiency, Pyridoxine-Dependent Epilepsy, Cobalamin disorders.	Pharmacological doses of biotin, pyridoxine (B6), hydroxycobalamin (B12).	Prevents seizures, metabolic strokes, and developmental delay; urgent intervention needed.	[169–171];
**ERT** Exogenous enzyme administration.	Lysosomal storage disorder with systemic substrate accumulation.	Pompe Disease, MPS I (Hurler), MPS II (Hunter), Krabbe Disease.	Intravenous recombinant enzyme (e.g., alglucosidase alfa, laronidase).	Imperative. ERT must begin before irreversible cardiac, muscular, or CNS damage occurs for optimal outcome.	[172, 173]
**Novel Gene-Targeted Therapies** Disease-modifying genetic/cellular interventions.	Monogenic defect leading to loss of functional protein or toxic gain-of-function.	SMA, MLD, ALD.	ASOs (nusinersen), gene therapy (onasemnogene abeparvovec, atidarsagene autotemcel), HSCT.	Paradigm-shifting. Therapy efficacy is profoundly greater when administered pre-symptomatically; NBS is the critical enabler.	[174–178]
**Disorders of Partial/No Direct Therapy** Ethical debate on screening utility.	Severe, often neonatal-onset defects with limited treatment options (supportive care).	Neonatal Urea Cycle Disorders (e.g., CPS1 def.), Severe Mitochondrial Disorders, Peroxisomal Biogenesis Disorders.	Supportive care, liver transplant (for some UCDs), palliative protocols, clinical trials.	Enables genetic counseling, prevents diagnostic odyssey, allows for prompt crisis management, and facilitates research/trial enrollment.	[164, 179–181]

AA, Amino Acid; ALD, Adrenoleukodystrophy; ASO, Antisense Oligonucleotide; CNS, Central Nervous System; CPS1, Carbamoyl Phosphate Synthetase 1; ERT, Enzyme Replacement Therapy; HSCT, Hematopoietic Stem Cell Transplantation; IEMs, Inborn Errors of Metabolism; MPS I, Mucopolysaccharidosis Type I (Hurler Syndrome); MPS II, Mucopolysaccharidosis Type II (Hunter Syndrome); MLD, Metachromatic Leukodystrophy; MSUD, Maple Syrup Urine Disease; NBS, Newborn Screening; Phe, Phenylalanine; PKU, Phenylketonuria; SMA, Spinal Muscular Atrophy; UCDs, Urea Cycle Disorders

## The economic feasibility of advanced NBS

The economic implications of transitioning from traditional biochemical NBS to integrated genomic and multi-omic approaches represent a critical, yet often underappreciated, dimension of the implementation debate. While the clinical promise of these technologies is substantial, their financial sustainability and cost-effectiveness remain largely unproven in the population screening context, with profound implications for healthcare systems worldwide.

### Is WGS cost-effective as a first-tier screening tool?

A rigorous systematic review of health economic evidence for whole-exome and whole-genome sequencing, encompassing 130 studies published between July 2016 and May 2024, found that only five studies assessed these technologies in the context of prenatal, newborn, or healthy population screening [182]. The overwhelming majority of economic evaluations have focused on WGS as a diagnostic tool for symptomatic populations, rather than as a population-wide screening intervention for healthy newborns [182]. In diagnostic settings, the evidence is more encouraging: a meta-analysis of cost-effectiveness studies estimated the pooled incremental net benefit of WGS over WES at I$4,073 (95% CI: I$2,426–I$5,720) and over chromosomal microarray at I$6,003 (95% CI: I$2,863–I$9,143) for affected infants and children [183]. The GEMINI study, which compared rapid genome sequencing (rGS) to a targeted 1,700-gene panel in 400 hospitalized infants, found that although rGS had a higher upfront cost ($12,297 vs. $2,449), it saved an estimated $158,592 per patient over the first year (95% CI: $63,701–$253,292) by reducing the need for additional diagnostic testing and shortening hospital stays [184, 185]. However, as the UK NIHR concluded in its comprehensive evidence synthesis, “the cost-effectiveness of WGS in a NBS context is unknown” [140]. None of the 86 economic evaluations identified in their systematic review were set in a screening context [140]. This evidence gap is profound: the economic calculus for screening healthy populations differs fundamentally from that for diagnosing symptomatic infants, as the former must balance the costs of testing millions of newborns against the benefits of preventing a relatively small number of rare but severe outcomes, all while accounting for the downstream costs of confirmatory testing, surveillance, and potential overdiagnosis [186]. The methodological challenges are substantial, including difficulties in identifying appropriate comparators, gaps in understanding long-term health outcomes and costs, and limited data on the effects of screening independent of improvements in treatment over time [186].

### Projected costs of integrating genomics and metabolomics

The integration of multiple omic technologies into a unified NBS platform presents a complex and potentially prohibitive cost structure. Current MS/MS-based screening programs operate at remarkably low per-infant costs, typically ranging from $50 to $150 per baby in high-income countries, including laboratory testing, follow-up, and program administration [187]. By contrast, the per-sample cost of WGS remains substantially higher, with estimates varying widely depending on sequencing depth, throughput, and infrastructure requirements. While sequencing costs have declined dramatically, the additional expenses associated with bioinformatic analysis, data storage, variant interpretation, and confirmatory testing create a total cost of ownership that far exceeds the raw sequencing price. The BeginNGS platform, designed specifically for genomic NBS, has been developed with the explicit goal of addressing cost and scalability challenges; a pilot trial demonstrated a true positive rate of 4.2% and sensitivity of 83%, with a positive predictive value of 100%. The ongoing BeginNGS adaptive, international clinical trial is prospectively comparing the utility, acceptability, feasibility, and cost-effectiveness of genomic NBS against standard biochemical screening [188, 189]. For next-generation metabolomics, the cost landscape is even less defined. Untargeted high-resolution mass spectrometry platforms require expensive instrumentation, specialized expertise, and sophisticated data analysis pipelines that are not yet standardized for population-scale screening [20]. The NewbornsInSA study in South Australia is one of the first prospective trials designed to assess the diagnostic yield, accuracy, and costs of an integrated multi-omic NBS model combining whole-genome sequencing and untargeted metabolomics [190]. However, until such studies report their findings, the true economic burden of multi-omic integration remains speculative. Crucially, any cost analysis must account for the cascade of downstream expenditures triggered by genomic screening: confirmatory biochemical testing, specialist consultations, serial monitoring, and, for infants with actionable findings, the potentially astronomical costs of gene-targeted therapies that can exceed two million dollars per patient [186]. These expenses are not optional adjuncts but integral components of a functional screening program, and their cumulative impact on healthcare budgets must be transparently evaluated before widespread implementation.

### Which healthcare systems can realistically implement these technologies?

The feasibility of implementing advanced genomic and multi-omic NBS varies dramatically across healthcare systems, shaped by existing infrastructure, workforce capacity, financial resources, and population health priorities. In high-income countries with centralized public health laboratory networks and established genomics infrastructure—such as the United Kingdom’s National Health Service, which has already integrated WGS into routine clinical care for rare diseases and cancer through the Genomic Medicine Service [182]—the pathway to genomic NBS is more navigable. Pilot programs like the Generation Study in England, the GUARDIAN study in New York, and the BabyDetect pilot in Belgium are actively demonstrating feasibility, diagnostic yield, and parental acceptability [191–193]. The BabyScreen+ study in Australia, which offered genomic screening to 1,000 newborns, showed that the approach is feasible and positively received by families, leading to molecular diagnoses in 1.6% of babies [194]. However, even in resource-rich settings, significant barriers remain: the current newborn screening system lacks capacity to meet growing demand, with challenges including an unprepared workforce, insufficient bioinformatic infrastructure, and the need for scalable models to handle the data volume and interpretative complexity of genomic screening [125]. For low- and middle-income countries (LMICs), implementing high-cost genomic NBS is considerably more remote. The World Health Organization has called for community genetics programs in LMICs focusing on cost-effective, context-appropriate interventions for congenital disorders [195]. Population-specific targeted panels have been estimated to cost as little as $10 for 10 Mendelian disorders, offering a transformative approach for resource-limited settings [196]. However, this vision requires substantial investment in laboratory capacity, workforce training, and healthcare infrastructure that remains out of reach for many nations. The BeginNGS trial’s international design explicitly aims to generate “equitable evidence to support broad implementation of gNBS,” recognizing that cost-effectiveness analyses must account for diverse healthcare contexts and resource constraints [189]. A fundamental tension exists: technologies offering the greatest promise for precision medicine are also those most likely to exacerbate global health disparities if implemented without deliberate attention to equity, affordability, and sustainability [186]. Policymakers must weigh the opportunity costs of investing in high-cost genomic screening against proven, cost-effective interventions that could save more lives per dollar spent, particularly where basic NBS for congenital hypothyroidism and phenylketonuria remains inconsistently available.

## The imperative for short-term reanalysis in multi-omic NBS

A critical yet underappreciated challenge is the substantial gap between the theoretical promise of multi-omic technologies and the current reality of evidence generation; for most conditions, evidence is insufficient and often unavailable for screening policy [93, 140]. Evidence from clinically detected cases is often not generalizable to screening populations, where less severe phenotypes are more common [141]. Reanalysis is imperative, yielding new diagnoses through gene discovery and database updates [197, 198], with incremental yields of 4.2% in ill children [199], 2.9% in epilepsy [200], and 20% in NBS cohorts [201].

Mechanisms for effective reanalysis include periodic querying of raw sequencing data using automated tools, tracking updates in clinical databases, and partially automated pipelines integrating clinical natural language processing of electronic health records [202, 203]. A feasible timeframe for reanalysis appears to be 2–2.5 years, though clinical symptom development should trigger immediate reanalysis [200]. For multi-omic NBS, the reanalysis imperative extends beyond genomics to metabolomics, as reference databases expand and new biomarkers are discovered [188]. Infants with initially “negative” or “uncertain” results must not be abandoned. A systematic reanalysis program is essential to ensure that multi-omic screening benefits all newborns, not just those with immediately actionable findings [201].

## Navigating the new frontier: ethical, logistical, and equity challenges

### Ethical and social implications (ELSI)

The shift to genomic NBS introduces profound ethical and social complexities. Traditional public health screening models are challenged by WGS, which necessitates a robust, opt-in informed consent process due to its potential to reveal adult-onset risks and carrier status. The uniquely identifiable and predictive nature of genomic and metabolomic data demands stringent cybersecurity and unambiguous policies governing data ownership, use, and future re-contact. Furthermore, identifying at-risk infants creates a “patient in waiting” phenomenon, imposing significant psychological burdens and anxiety on families for conditions that may never manifest [179, 204, 205].

### The equity imperative

The promise of advanced NBS carries a significant risk of exacerbating global health inequities. A primary obstacle is the “genomics gap,” where reference databases dominated by European-ancestry data cause higher rates of uninterpretable variants and missed diagnoses in underrepresented populations, embedding bias into precision medicine [206–208]. Furthermore, identifying a disorder like Pompe disease is futile without guaranteed access to prohibitively expensive therapies and specialized care, creating an unethical “screen-and-abandon” dilemma [59, 60, 149–151]. This high-tech paradigm is largely inaccessible in low- and middle-income countries (LMICs), where public health strategy must prioritize cost-effective screening for treatable conditions like congenital hypothyroidism [209]. While simplified, targeted genetic tools could have a future role in LMICs, the fundamental imperative is to ensure that technological advancement does not widen the chasm of healthcare disparity but is instead guided by a commitment to equitable implementation and access for all newborns.

### System logistics and workforce

**Fig. 3:**
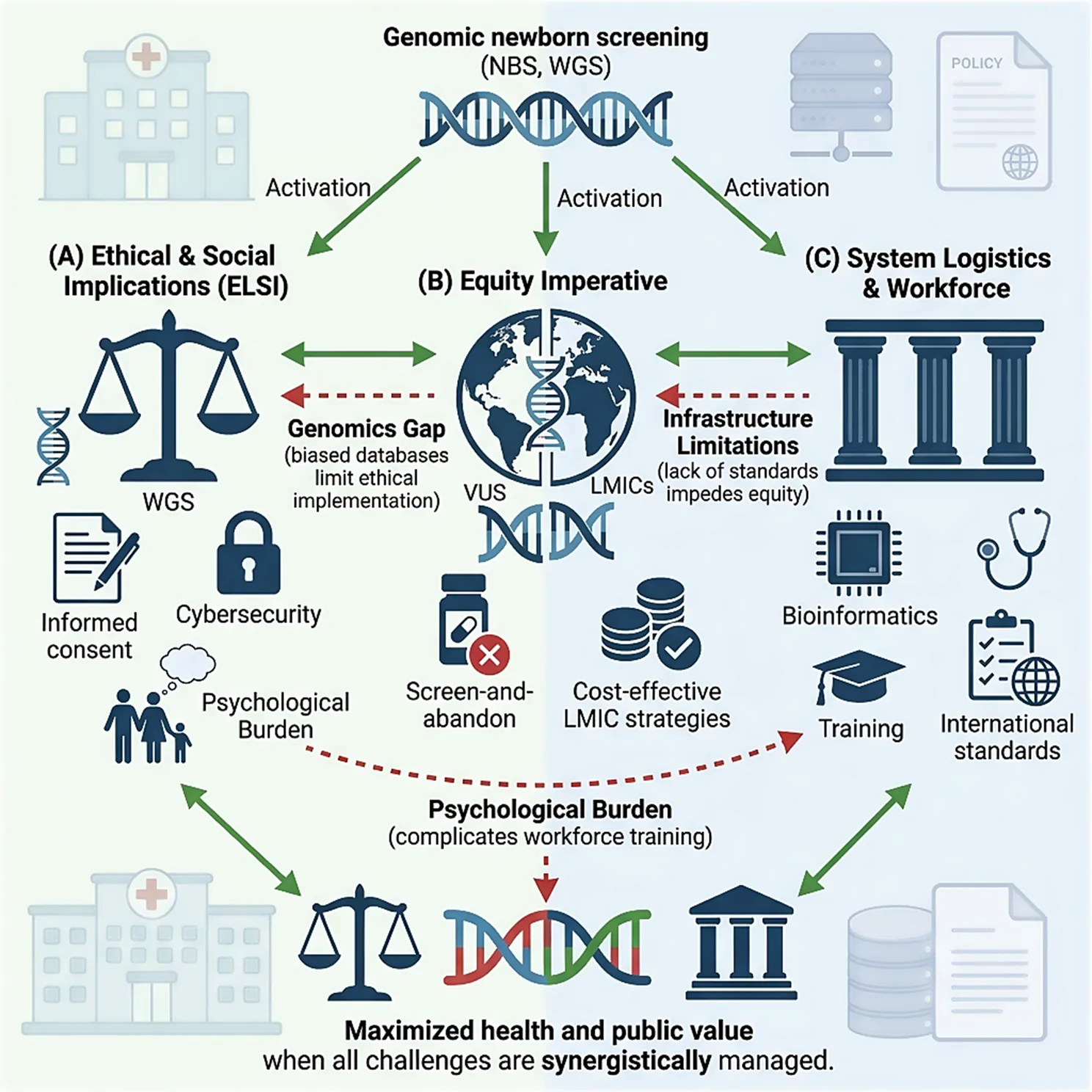
Navigating the new frontier: a conceptual map of the ethical, equity, and logistical challenges in advanced newborn screening. The figure illustrates three primary constraint domains that must be addressed to realize the promise of genomic and multi-omic newborn screening. (**A**) Ethical & social implications (ELSI): represented by scales of justice, this domain highlights the need for robust informed consent models for genomic data, stringent cybersecurity for uniquely identifiable information, and management of the psychological “patient in waiting” burden on families. (**B**) The equity imperative: represented by a divided globe, this domain underscores the risk of exacerbating health disparities through biased genomic databases (“the Genomics gap”), the unethical “screen-and-abandon” dilemma when therapies are inaccessible, and the need for context-appropriate, cost-effective strategies in low-resource settings. (**C**) System logistics & workforce: represented as foundational pillars, this domain outlines the essential infrastructural needs: major investment in bioinformatic capacity, specialized interdisciplinary training for clinicians and counselors, and the development of harmonized international standards for data and variant reporting. Arrows denote the critical interdependence of these challenges. Abbreviations: NBS, newborn Screening; WGS, whole genome Sequencing; VUS, variant of uncertain Significance; LMICs, low- and middle-income countries. Image was created using BioRender, in conjunction with Microsoft PowerPoint for final assembly and labeling

Successful implementation of integrated multi-omic NBS requires substantial investment in bioinformatic infrastructure, comprehensive workforce training for interpreting complex results, and harmonized technical, reporting, and variant classification standards across regions and laboratories [210–212]. As illustrated in Fig. 3, this transition is constrained by a triad of interdependent challenges: profound ELSI concerns, systemic risks to health equity, and formidable logistical and workforce requirements.

## Global perspectives on NBS implementation

The implementation of NBS for IEMs varies enormously across regions, shaped by local epidemiology, healthcare infrastructure, and economic resources. While high-income countries have largely established comprehensive MS/MS-based programs, LMICs face unique challenges and have developed diverse strategies offering valuable lessons for global screening expansion.

### Asia: diverse implementation models

The Asia-Pacific region presents a spectrum from well-established national programs to nascent initiatives. China has made remarkable progress: a 19-year Shanghai report documented screening of 1.17 million newborns [213], a Changzhi study screened 153,956 infants [214], and Xinjiang screened 107,741 newborns [215], demonstrating population-scale MS/MS feasibility. Cost-effectiveness analyses confirm MS/MS is attractive at current willingness-to-pay thresholds [187]. Japan pioneered LSD screening, with a recent study screening 733,838 newborns and diagnosing 101 cases, including 75 with Fabry disease [216]. Thailand offers a model for rural implementation: the KKU-IEM platform covered 98.6% of 123,692 live births, identifying 101 CH cases (1:1,208) and 20 IEM cases (1:6,100), with IEM incidence double that of Bangkok, highlighting the importance of regional data [217]. India, with ~67,385 daily births, faces daunting challenges, yet NGS and bioinformatics advancements offer potential [218]. Korea’s NewbornSeq combines biochemical tests with a 97-gene panel; 90% of newborns undergo screening for ~70 disorders, and WGS integration is underway [219, 220].

### Africa: the underserved continent

Africa remains the continent with the most significant NBS gaps. Despite WHO recommendations, universal NBS has not been accomplished, as communicable diseases historically took priority [221]. A 2025 situation analysis highlights a pivotal opportunity: existing HIV/AIDS and immunization infrastructure and DBS sampling could provide platforms for NBS integration [221]. Nigeria has few screening programs, lacks epidemiological data across six geopolitical zones, and faces insufficient facilities and low provider awareness. Private panels (15,600–150,000 NGN) are inaccessible to most. A 2025 Abuja meeting appealed to add CH to sickle cell screening [222]. South Africa has limited implementation despite clinical guidelines; NBS is not a health priority, and no legislation exists for testing, though the Potchefstroom Laboratory for Inborn Errors of Metabolism (PLIEM) has operated since 1983 (https://natural-sciences.nwu.ac.za/centre-human-metabolomics/pliem).

### Latin America: progress and persistent disparities

Latin America shows highly heterogeneous progress; ~15 of 20 countries have NBS programs, but coverage varies widely, remaining below 70% in some countries like Mexico [223]. Brazil has the most comprehensive program: screening began in 1976 for PKU, expanded to CH in 1980, and was formally incorporated into national policy in 1990. The PNTN launched in 2001, achieving 80% coverage by 2005 [223]. A landmark 2021 law mandated expansion to include treatable IEMs, SCID, and SMA—the first in Latin America to include mandatory MS/MS-based screening. However, regional disparities persist: states like São Paulo show high coverage, while Northern regions lag [223–225]. A Porto Alegre pilot explored expanded NBS for IEMs via MS/MS [225]. Colombia established reference values for amino acids, acylcarnitines, and succinylacetone [226, 227].

### Comparative implementation strategies

Several key insights emerge: high-income Asian countries like Japan and Korea have pioneered LSD and genomic integration; middle-income countries like China and Brazil demonstrate scale-up feasibility with political commitment; lower-middle-income countries like Thailand and India show targeted, phased implementation can succeed but require sustained investment; low-income African nations face fundamental barriers, lack of epidemiological data, insufficient diagnostics, limited awareness, and competing priorities [228]. A systematic review underscores that precise prevalence and incidence estimates in LMICs are needed to advocate for NBS expansion [228]. As one analysis noted, “Newborn screening is not a health priority in South Africa and no legislation or even recommendations exist for testing” [229], a sentiment echoing across much of the continent.

## Global policy differences in NBS implementation

NBS policies vary substantially across countries, shaped by epidemiological, financial, and healthcare infrastructure differences [230]. Within the European Union, great disparities exist: Italy screens for 48 conditions, Austria for 29, and Romania for just 4, creating a concerning “postcode lottery” where a child’s health outcomes depend on birthplace (https://share.google/MveXVqYJC39ChNwYs). The ERDERA initiative, funded with €380 million under Horizon Europe, aims to eliminate these disparities through coordinated screening expansion (https://erdera.org/article/harmonizing-european-newborn-screening/). In federal systems like the United States, the Recommended Uniform Screening Panel (RUSP) provides guidelines, but only 52% of babies are born in states that have adopted full RUSP alignment (https://newbornscreening.hrsa.gov/about-newborn-screening/recommended-uniform-screening-panel). Similarly, Canada and Australia operate state-based programs with regional differences creating inequities in care access [231, 232]. The most profound divide exists between high-income countries (HICs) and low- and middle-income countries (LMICs). NBS is well-established in most HICs but remains limited in LMICs, despite WHO recommendations for universal adoption (https://www.who.int/publications/i/item/9789290211389). Approximately 94% of congenital anomalies occur in LMICs, yet these regions have the least developed screening infrastructure [221]. The 77th World Health Assembly in 2024 passed a resolution calling for NBS in LMICs, signaling growing global commitment [233]. International collaborations comparing programs across Saudi Arabia, Japan, Singapore, Canada, Australia, the UK, and the US highlight both variations and shared challenges across diverse healthcare systems [230].

## Data-sharing governance frameworks for NBS

The integration of genomics and multi-omics into NBS generates vast, uniquely identifiable datasets that necessitate robust governance frameworks to balance data sharing for research with privacy protection and equitable benefit-sharing [234]. Unlike traditional clinical data, genomic information is inherently identifiable, permanent, and predictive, creating privacy risks that persist across an individual’s lifetime [235]. Traditional NBS operates under public health mandates with opt-out consent, but genomic screening demands more nuanced consent models such as tiered consent, dynamic consent, or broad consent with independent oversight [236, 237]. The BabySeq Project demonstrated that 75.7% of approached families consented to receive genomic results when supported by robust informed consent, suggesting that families are willing to engage with detailed consent processes [238].

International data sharing is essential for training AI algorithms on diverse populations to address the “genomics gap” where underrepresented groups are disadvantaged by biased reference databases [239]. However, cross-border data exchange faces legal barriers, including the European Union’s General Data Protection Regulation (GDPR), which imposes strict requirements for data transfer and grants individuals rights to access and delete their data (https://eur-lex.europa.eu/eli/reg/2016/679/oj/eng). Initiatives like the European 1+ Million Genomes (1+MG) initiative and the Global Alliance for Genomics and Health (GA4GH) are developing interoperable frameworks for secure genomic data sharing that respect jurisdictional differences while enabling collaborative research (https://digital-strategy.ec.europa.eu/en/policies/1-million-genomes) [240]. Effective governance requires oversight bodies comprising diverse stakeholders like clinicians, scientists, ethicists, patient advocates, and community representatives, with transparent data use policies, clear protocols for returning incidental findings, and robust public engagement to build and maintain trust [125].

## Future perspectives

The future trajectory of NBS for IEMs is firmly towards an intelligent, multi-tiered system. This adaptive framework begins with a primary high-throughput screen using robust MS/MS and targeted DNA panels for common, treatable disorders. Ambiguous results would automatically trigger reflexive second-tier analysis on the same sample, employing deeper genomic sequencing or broad metabolomic profiling. A central, continuously learning AI engine would underpin the entire process, triaging data, integrating multi-omic streams, and generating diagnostic probabilities to guide urgent action [142, 143]. However, given the substantial gaps in evidence generation and the slow pace of database updates, a mandatory short-term reanalysis system must be established to periodically re-evaluate initially “negative” or “uncertain” cases, “rescuing” those that would otherwise remain undiagnosed [93, 140, 241]. Furthermore, the recognition that phenotypic variability in IEMs is modulated by genetic background, environmental factors, and modifier genes necessitates the eventual incorporation of polygenic risk scores to refine risk stratification beyond binary variant classification. Crucially, the initial screening result would become the foundational entry in a secure, longitudinal health record, informing a child’s lifelong medical care while contributing anonymized data to research repositories. This integrated vision transforms NBS from a singular test into a dynamic, learning component of precision pediatric medicine, but its success depends on equitable global implementation, sustained investment in infrastructure and workforce, and continuous evidence generation to ensure that benefits outweigh risks for all newborns [1, 4, 12, 72, 108, 118, 145, 175, 177, 208].

## Conclusion

The new frontier of newborn screening is no longer defined by a single technology. It is defined by the synergistic integration of genomics, metabolomics, and data science. However, this powerful integration demands an equally sophisticated commitment to addressing the critical challenges that accompany it: incomplete penetrance, overdiagnosis, algorithmic bias, evidence gaps, and the profound disconnect between rapid data generation and slow knowledge translation. The establishment of mandatory short-term reanalysis systems is essential to rescue initially undiagnosed cases, while the incorporation of polygenic risk scores may refine risk stratification beyond binary variant classification by accounting for genetic and environmental modifiers. Furthermore, the economic feasibility of these technologies remains unproven in population screening contexts, and global disparities in access threaten to exacerbate rather than alleviate health inequities. The goal is not to screen for everything possible, but to build a smart, just, and actionable system that ensures every newborn has the best possible start in life, turning the promise of pre-symptomatic medicine into a universal reality. The journey from a spot of blood on a card to a comprehensive biological forecast has begun, and it will redefine pediatric medicine for the 21st century, provided that technological advancement is guided by ethical responsibility and a commitment to leaving no child behind.

## Data Availability

No datasets were generated or analysed during the current study.

## Abbreviations

ACMG: American College of Medical Genetics and Genomics
AI: Artificial Intelligence
ALD: Adrenoleukodystrophy
CH: Congenital Hypothyroidism
DBS: Dried Blood Spot
HER: Electronic Health Record
ELSI: Ethical, Legal, and Social Implications
ERT: Enzyme Replacement Therapy
GA4GH: Global Alliance for Genomics and Health
GDPR: General Data Protection Regulation
HICs: High-Income Countries
HR-MS: High-Resolution Mass Spectrometry
IEMs: Inborn Errors of Metabolism
LMICs: Low- and Middle-Income Countries
LSDs: Lysosomal Storage Diseases
MCADD: Medium-Chain Acyl-CoA Dehydrogenase Deficiency
ML: Machine Learning
MLD: Metachromatic Leukodystrophy
MPS I: Mucopolysaccharidosis Type I
MS/MS: Tandem Mass Spectrometry
MSUD: Maple Syrup Urine Disease
NBS: Newborn Screening
NGS: Next-Generation Sequencing
NLP: Natural Language Processing
NMR: Nuclear Magnetic Resonance
PKU: Phenylketonuria
RUSP: Recommended Uniform Screening Panel
SCID: Severe Combined Immunodeficiency
SMA: Spinal Muscular Atrophy
VUS: Variant of Uncertain Significance
WES: Whole Exome Sequencing
WGS: Whole Genome Sequencing
WHO: World Health Organization
